# A simple DNA gate motif for synthesizing large-scale circuits

**DOI:** 10.1098/rsif.2010.0729

**Published:** 2011-02-04

**Authors:** Lulu Qian, Erik Winfree

**Affiliations:** 1 Bioengineering, California Institute of Technology, Pasadena, CA 91125, USA; 2 Computer Science, California Institute of Technology, Pasadena, CA 91125, USA; 3 Computation and Neural Systems, California Institute of Technology, Pasadena, CA 91125, USA

**Keywords:** nucleic acids, synthetic biology, strand displacement circuits, molecular programming

## Abstract

The prospects of programming molecular systems to perform complex autonomous tasks have motivated research into the design of synthetic biochemical circuits. Of particular interest to us are cell-free nucleic acid systems that exploit non-covalent hybridization and strand displacement reactions to create cascades that implement digital and analogue circuits. To date, circuits involving at most tens of gates have been demonstrated experimentally. Here, we propose a simple DNA gate architecture that appears suitable for practical synthesis of large-scale circuits involving possibly thousands of gates.

## 1. Introduction

RNA- and DNA-based catalysts [1–3] and logic gates [4–6] have been proposed as general-purpose components for synthesizing chemical circuits [6–8] with applications in medical therapeutics [9], nanotechnology [10] and embedded control of chemical reactions [11]. Progress in this direction will depend upon advances in three areas: (i) developing input/output interfaces between the DNA circuits and biomedically relevant molecules [12], DNA nanomachines [13,14], and general chemistries [15,16]; (ii) developing DNA circuit construction techniques that scale up so that large and interesting circuits can be systematically created [6,8,17,18]; and (iii) extending the DNA programming methodology beyond well-mixed solutions to include spatial structures at the molecular [19–21] and macroscopic scales [22–24].

In this paper, we focus on the second challenge, using DNA strand displacement cascades [3,6,17,18]. We introduce a DNA gate motif suitable for scaling up to large circuits, along with an abstract circuit formalism that aids the design and understanding of circuit behaviour. To illustrate the rich potential of this approach, we show how to implement arbitrary feedforward digital logic circuits, arbitrary relay circuits and analogue circuits exhibiting a variety of temporal dynamics. Large circuits can be made fast and reliable because first, the gates can act catalytically to amplify small signals propagating through the circuit, and second, the gates can incorporate a threshold to clean up noise and erroneous signals. Further, thanks to the modular design of the gate motif, systematic construction can be automated by a straightforward compiler that converts a high-level description of a circuit function into a molecular implementation at the level of DNA sequences. Finally, we argue that synthesis and preparation of molecular components can be parallel and scalable. Our estimates, based on the available sequence design space for typical DNA lengths, current synthesis technologies using parallel DNA microarrays, and plausible mass-action rates and concentrations, suggest that circuits involving thousands of distinct gates may be designed, synthesized and executed.

## 2. A simple DNA gate motif

**Figure 1.**
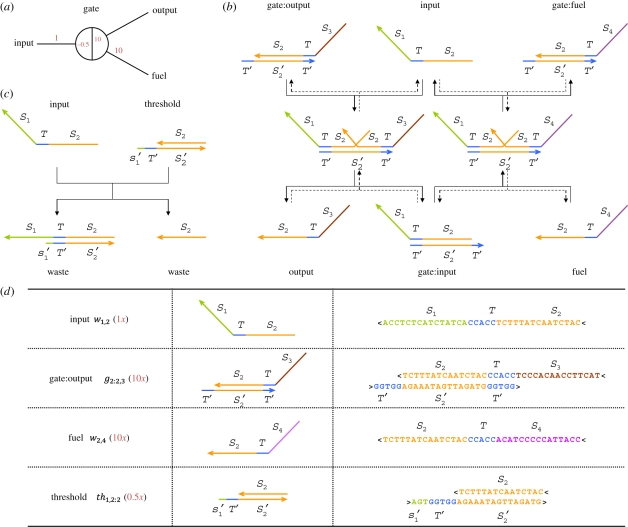
The DNA motif for ‘seesaw’ gates. (*a*) Abstract gate diagram. Red numbers indicate initial concentrations. (*b*) The DNA gate motif and reaction mechanism. *S*_1_, *S*_2_, *S*_3_ and *S*_4_ are the recognition domains; *T* is the toehold domain; *T*′ is the Watson–Crick complement of *T*, etc. Arrowheads mark the 3′ ends of strands. Signal strands are named by their domains from 3′ to 5′, i.e. from left to right, so the input is *S*_1_*TS*_2_; gate base strands and threshold bottom strands are named by their domain from 5′ to 3′. All reactions are reversible and unbiased; solid lines indicate the dominant flows for the initial concentrations shown in (*a*), while the reverse reactions are dotted. (*c*) The threshold motif and reaction mechanism. The toehold is extended by a few bases (*s*′_1_, the complement of the first few 5′ bases of *S*_1_), providing an increased rate constant relative to the gate itself. Branch migration intermediate states are omitted from the diagram. (*d*) Example sequences. Gate complexes and signal molecules are shown at the domain level (second column) and at the sequence level (third column). Here, recognition domain sequences are 15 nt, the toehold domain sequence is 5 nt, and the toehold is extended by 3 nt for the threshold. Other lengths are possible, so long as they ensure that recognition domains will not spontaneously dissociate, toehold exchange is fast, and thresholding is sufficiently faster.

We begin by describing the elementary building block for our circuits. Figure 1*a* shows the abstract diagram of a single gate, configured so that an ‘input’ catalytically converts ‘fuel’ to ‘output’ when the input concentration exceeds a threshold level. Signals on the wires, e.g. input, fuel and output, will correspond to single-stranded DNA molecules we call ‘signal strands’, while the gate node itself will correspond to partially double-stranded DNA molecules we call ‘gate:signal complexes’ and ‘threshold complexes’. The catalytic cycle is achieved by a series of interactions between signal strands and gate complexes, based on the underlying mechanism of ‘toehold-mediated DNA strand displacement’ [25] in which a single-stranded DNA molecule displaces another from a double-stranded complex with the help of a short ‘toehold’ domain.

Signal strands have a uniform format consisting of a left ‘recognition domain’, a central ‘toehold’, and a right recognition domain (figure 1*b*). In our example, the input is *S*_1_*TS*_2_, the output is *S*_2_*TS*_3_ and the fuel is *S*_2_*TS*_4_. Recognition domain *S*_2_ is the active domain participating in the catalytic reactions associated with this gate, while *S*_1_, *S*_3_ and *S*_4_ can similarly react with other gates. The recognition domains are relatively long (e.g. 15 nt) to ensure stable hybridization, while the toehold is relatively short (e.g. 5 nt) to ensure fast release of strand displacement products, but still long enough to effectively initiate strand displacement. While many distinct recognition domains may be used, all molecules use the same ‘universal’ toehold sequence; identity is conferred by the recognition domain sequences. The structure and mechanism of the gate are intrinsically symmetric, so that labels ‘input’, ‘output’ and ‘fuel’ refer only to roles played by the signal strands in this asymmetrically configured example.

A gate is represented abstractly as a two-sided node with one or more wires connected to each side. A signal strand may either be free in solution with its toehold exposed, indicating activity on a wire, or else bound to a gate ‘base strand’ (e.g. the bottom strand *T*′*S*′_2_*T*′ of the gate:output complex) with its toehold sequestered and thus inactive. Because of their DNA implementation, gates have an intrinsic polarity that distinguishes the two sides, which we will refer to as the ‘left side’ and the ‘right side’. Wires must connect the right side of one gate to the left side of another. (For example, the output strand *S*_2_*TS*_3_ uses subsequence *S*_2_*T* to react with one gate from the right side and subsequence *TS*_3_ to react with another gate from the left side.) Gate:signal complexes also have a uniform structure. The gate base strand consists of a recognition domain identifying the gate, flanked by two toehold domains, and is always complexed with a signal strand facing either left or right; only one toehold is exposed at any given time. In our example, the gate:output complex comprises gate base strand *T*′*S*′_2_*T*′ bound to signal strand *S*_2_*TS*_3_ facing right (figure 1*b*). In the following, ‘signal strand’ will mean free signal strand, unless otherwise specified.

A threshold may also be associated with a gate, absorbing a signal strand at a faster rate compared to the gate. Threshold complexes are similar to gate complexes, but with an extended toehold on one side and no toehold on the other side—consequently, the top strand has no toehold and is therefore inert when released. The toehold is extended by a few nucleotides (e.g. 3 nt) to ensure faster reaction rates [26]. In our example, the threshold complex that absorbs input *S*_1_*TS*_2_ comprises bottom strand *s*′_1_*T*′*S*′_2_ and top strand *S*_2_ (figure 1*c*).

Each position within the abstract diagram (figure 1*a*) corresponds to a specific molecule, and each red number in that position indicates the (relative) initial concentration for that molecule: positions on each wire correspond to free signal strands, while positions within the node at the end of each wire correspond to gate:signal complexes (positive numbers) or threshold complexes (negative numbers).

Gates support three elementary behaviours. ‘Stoichiometric triggering’ occurs when a free signal strand (e.g. input) binds to the exposed toehold of a gate complex (e.g. gate:output), initiating branch migration and the subsequent release of the signal strand previously sequestered in the gate complex (e.g. output; figure 1*b*, left pathway). Thus, ‘toehold exchange’ [27] has been effected; the resulting gate complex (e.g. gate:input) now has a toehold exposed on the opposite side. Consequently, the overall reaction is reversible: the released signal strand can bind to the gate again, triggering the release of the original signal strand. Overall, stoichiometric triggering allows the exchange of equal amounts of activity from a wire on one side of a gate to a wire on the other side. This back-and-forth motion is the inspiration for our name for this motif: ‘seesaw gates’.

If fuel is present, then a ‘catalytic cycle’ may occur wherein after stoichiometric triggering by the input to produce the output, fuel may bind to the gate and displace the input, freeing it to trigger more release of the output (figure 1*b*, both pathways). This cycle, a simplification of Zhang *et al.* [3] that was demonstrated in Zhang & Winfree [27], allows an arbitrarily small amount of input, over time, to catalyse the release of an arbitrarily large amount of output. Because there is also a reverse action whereby the output (rather than fuel) displaces the gate-bound input, as well as an action whereby the input displaces gate-bound fuel (rather than gate-bound output), we expect a single isolated gate to establish an equilibrium rather going to completion. Critically, if no input is present, there is no fast pathway for the fuel to directly displace the output; strand displacement without a toehold is many orders of magnitude slower [26–28], and we neglect it here.

Finally, ‘thresholding’ occurs whenever a threshold complex is present (figure 1*c*). Due to the threshold complex's extended toehold, and the exponential dependence of reaction rate on toehold length [26,27], input strands will react with threshold complexes faster than they react with gate complexes—we assume a 20-fold speed-up in this paper. The two resulting ‘waste’ complexes are inert, as they have no exposed toehold domains. Consequently, only if the input concentration exceeds the threshold concentration, can the excess input partake significantly in stoichiometric triggering or catalysis. With subthreshold input levels, there will still be a small ‘leak’ due to inputs that react with gates before encountering a threshold. Fortunately, even leaky thresholding behaviour can enable reliable circuit function by cleaning up after upstream leaky reactions.

It is worth considering the driving forces for these three behaviours. Previous strand displacement circuits primarily made use of additional base pairing [6] or exploited the release of additional molecules [3] to drive reactions forward, but neither mechanism is essential in seesaw gate circuits. Stoichiometric triggering involves a reversible reaction with the same number of molecules and the same number of base pairs in the reactants as in the products, so the standard free energy difference is approximately zero. Thus, the driving force comes from the entropic free energy of concentration imbalances, which encourages equalization. The catalytic cycle is similarly driven exclusively by entropic free energy, but introduces an auxiliary species, the fuel, whose initial concentration can serve as an energy source to drive the reaction in a desired direction. On the other hand, thresholding involves an essentially irreversible reaction with a net gain of base pairs (8 bp in our example) and thus a significant standard free energy change. A downstream threshold can therefore act as a drain on an upstream entropy-driven reaction, further pulling the reaction forward.

The single-gate circuit shown in figure 1*a* is configured for catalysis with thresholding. The diagram specifies the gate:output complex, fuel signal strand, input signal strand and threshold complex with respective concentrations 10*x*, 10*x*, 1*x* and 0.5*x*, where 1*x* is a standard concentration, perhaps 50 nM. With these initial concentrations, the input strand will first overcome the threshold and then act catalytically to facilitate the equilibration of the output strand and the fuel strand to approximately 5*x* each. This level can be estimated by noting that by symmetry the two wires will have similar activity at equilibrium, while the total concentration on each wire and the total concentration within the gate remain constant at 10*x* each. Thus, a single seesaw gate can robustly amplify an input signal that exceeds a threshold. Other behaviours, including stoichiometric triggering, are possible with different configurations of wires and concentrations. When connected into circuits involving many interacting seesaw gates, complex functional behaviour can be obtained.

## 3. Abstract circuit formalism

The abstract network representation introduced in figure 1*a* facilitates concise reasoning about circuits involving many interacting gates. In general, a circuit consists of a number of gate nodes and a number of wires between gate nodes. Each gate node consists of a left side and a right side, and it may connect to any number of wires on each side (figure 2*a*). Each wire connects exactly two gates (figure 2*b*), from the left side of one to the right side of the other. Some wires, such as input, output and fuel, may connect to ‘virtual gates’ whose total base strand concentration is zero. Virtual gates provide a consistent naming scheme for wires that appear connected on just one side, making it easy to extend a circuit or compose circuits together. For clarity, gates with non-zero base strand concentration can be referred to as ‘realized gates’. A seesaw circuit then consists of a number of gate nodes connected by wires between their left and right sides (figure 2*c*). The diagram can be annotated to indicate the initial state of the circuit (figure 2*a*).

**Figure 2.**
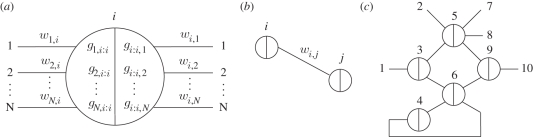
Abstract diagrams for seesaw gate circuits. (*a*) The general form of a gate node. Each gate *i* may be connected to many wires on each side, potentially all *N* nodes in the network, including itself. For each wire from the right side of gate *i* to the left side of gate *j*, the initial concentration of the free signal *w*_*i,j*_ may be written above the wire, and the initial concentrations of gate complex *g*_*j,i:i*_ (*w*_*j,i*_ bound to gate *i*) and *g*_*i:i,j*_ (*w*_*i,j*_ bound to gate *i*) may be written within the node at the ends of the corresponding wires. Gate concentrations are simply omitted if they are zero. Initial concentrations of *th*_*j,i:i*_ (the threshold for *w*_*j,i*_ arriving at gate *i*) and *th*_*i:i,j*_ (the threshold for *w*_*i,j*_ arriving at gate *i*) may be written in the same locations as *g*_*j,i:i*_ and *g*_*i:i,j*_, respectively, but as negative numbers—or omitted if they are zero. (*b*) The general form of a wire. Each wire is specifically connected on its left end to the right side of a gate node, and connected on its right end to the left side of a gate node. (*c*) An example circuit with five realized gates (numbered circles), five virtual gates (numbers at ends of wires), and 11 wires. Each wire is identified by the two gates it connects; thus the virtual gates serve to provide full names (and sequences) to their incident wires. Note that circuit diagrams may be drawn without providing gate numbers, as they are not relevant to circuit function.

The implementation of any such circuit diagram using DNA molecules is straightforward. A circuit with *N* (realized or virtual) gates requires a single universal toehold sequence *T* and a set of *N* sufficiently distinct *m*-mer sequences *S*_1_, *S*_2_, …, *S*_*N*_, one for each gate. The wire from the right side of gate *i* to the left side of gate *j* is called *w*_*i,j*_ and is implemented as a free signal strand with sequence *S*_*i*_*TS*_*j*_. The gate *i* base strand, *T*′*S*′_*i*_*T*′, is always part of a gate complex: *g*_*i:i,j*_ refers to the gate complex in which the base strand is bound to the left side of signal strand *S*_*i*_*TS*_*j*_, and *g*_*k,i:i*_ refers to the gate complex in which the base strand is bound to the right side of signal strand *S*_*k*_*TS*_*i*_. Note that *g*_*i:i,i*_ and *g*_*i,i:i*_ are both complexes of signal strand *S*_*i*_*TS*_*i*_ and gate *i* base strand *T*′*S*′_*i*_*T*′, but the former has the signal strand bound on its left side, leaving the gate's left toehold exposed, while in the latter case the signal strand is bound on its right side, leaving the gate's right toehold exposed. The threshold complex for wire *w*_*i,j*_ within gate node *j* is called *th*_*i,j:j*_ and is implemented as a complex of top strand *S*_*j*_ and bottom strand *s*_*i*_′*T*′*S*_*j*_′; on the other side of the same wire, *th*_*i:i,j*_ within gate node *i* also absorbs wire *w*_*i,j*_, and is implemented as *S*_*i*_ and *S*_*i*_′*T*′*s*_*j*_′.

Because all components are in a standard form, the set of chemical reactions modelling a seesaw circuit can be written concisely. The reversible toehold exchange steps, where a free signal strand displaces a bound signal strand from a gate complex, are modelled by3.1
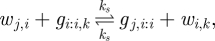
where *k*_*s*_ is a bimolecular rate constant and *i,j,k* ∈ {1, 2, …, *N*}. Similarly, the irreversible thresholding steps, where a signal strand is absorbed by a threshold complex in the gate node on either end of its wire, are modelled by3.2
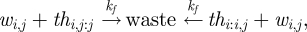
where *k*_*f*_ is a bimolecular rate constant much faster than *k*_*s*_. Using standard mass action chemical kinetics, this gives rise to a system of ordinary differential equations (ODEs) for the dynamics:3.3
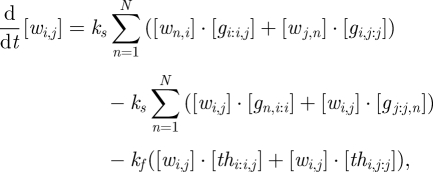
3.4
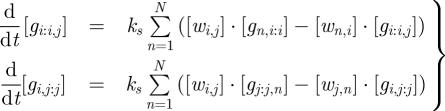
and3.5
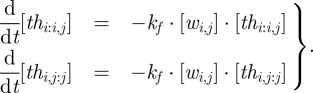


These dynamics have conserved quantities for each gate node and for each wire. First, the total amount of the gate *i* base strand *T*′*S*′_*i*_*T*′, whether its left toehold is covered ([*g*_1,*i:i*_] … [*g*_*N,i:i*_]) or its right toehold is covered ([*g*_*i:i*,1_] … [*g*_*i:i,N*_]), remains constant for all times *t*:3.6
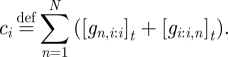


Second, the total amount of each signal strand *S*_*i*_*TS*_*j*_, whether free in solution ([*w*_*i,j*_]), bound in a gate complex ([*g*_*i:i,j*_] and [*g*_*i,j:j*_]), or absorbed by a threshold ([*th*_*i,j:j*_]_0_ − [*th*_*i,j:j*_]_*t*_ and [*th*_*i:i,j*_]_0_ − [*th*_*i:i,j*_]_*t*_), remains constant, so3.7
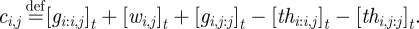


Note that we omitted the constant values [*th*_*i:i,j*_]_0_ and [*th*_*i,j:j*_]_0_ from this sum; *c*_*i,j*_ represents the amount by which the total signal strand concentration (in any form) exceeds the total threshold complex concentration. If there is more threshold than signal strand, then *c*_*i,j*_ could be negative; in that case, any activity on the wire will be immediately suppressed.

Additional constraints come from equilibrium, if and when it is obtained. Because threshold complexes inhibit activity on their wires until the threshold is overcome, and because equilibration of wires on one side of a gate node can depend upon activity in a wire on the other side, a seesaw circuit can settle down into a state with a complex pattern of active and inactive wires, with only certain gates and wires participating in the equilibrium. Consider, then, a network that has settled down in such a configuration, and treat concentrations arbitrarily near zero as exactly zero, for convenience. Consider further a gate node *i* that has at least one active wire on each side, undergoing active exchange with gate complexes. Equilibrium enforces a simple relationship between the free and bound forms of the signal strands, namely that their ratio with respect to a particular gate must be identical for all active wires connected to that gate. This follows immediately from equation (3.1) and the detailed balance equation *k*_*s*_· [*w*_*j,i*_] · [*g*_*i:i,k*_] = *k*_*s*_· [*g*_*j,i:i*_]· [*w*_*i,k*_]. To wit, for each gate *i*, at equilibrium all active wires achieve the ratio3.8
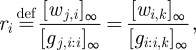
where *j* and *k* refer to any active wires on the left and right sides of the gate node.

As an example, we can calculate the equilibrium concentrations for a single realized gate, *i*, connected to a number of virtual gates, as in figure 1*a*. Without loss of generality, we assume that all wires have initial concentrations larger than their respective thresholds, i.e. all *c*_*i,j*_ are positive, and all thresholds will eventually be consumed. We can derive all equilibrium wire and gate concentrations from our knowledge of the constants *c*_*i*_ and *c*_*i,j*_, which can be calculated directly from the initial conditions. The equilibrium wire concentration [*w*_*i,j*_]_∞_ depends upon the gate ratio *r*_*i*_ and the initial concentrations on that wire, as can be derived from equations (3.7) and (3.8) and the definition of a virtual gate (i.e. [*g*_*i,j:j*_]_*t*_ = [*th*_*i,j:j*_]_*t*_ = 0 for virtual gate *j*):3.9
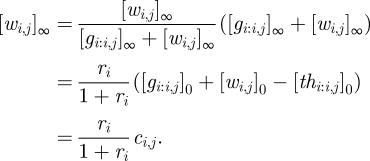


Symmetrically, where gate *k* is also a virtual gate:3.10
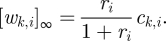


To solve for the equilibrium gate ratio *r*_*i*_, we can rewrite equation (3.6) using equation (3.8), to obtain:3.11
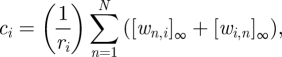
whereupon substituting in equation (3.9) immediately gives us3.12
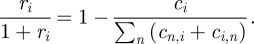


It is easy to work out the equilibrium wire concentrations directly from a diagram, such as figure 1*a*. We first calculate 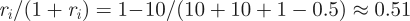, where the numerator is the sum of all non-negative red numbers within the gate node, and the denominator is the sum of all red numbers in the entire diagram. Then, the input [*w*_1,2_]_∞_ ≈ 0.5*x* × 0.51 ≈ 0.26*x*, where 0.5*x* is the excess of input over the threshold. Similarly, the output [*w*_2,3_]_∞_ ≈ 10*x* × 0.51 = 5.1*x*, where 10*x* is the sum of red numbers on the output wire, and likewise the fuel [*w*_2,4_]_∞_ ≈ 10*x* × 0.51 = 5.1*x*.

In conclusion, the abstract seesaw circuit formalism provides a precise ‘executable’ interpretation for arbitrary seesaw circuit diagrams, in terms of formal chemical reaction networks and their associated mass action ODEs. The uniformity of components in seesaw gate circuits also facilitates their analysis and simulation. We have written routines for concisely representing, constructing and simulating models of seesaw gate circuits in Mathematica. For efficient simulation of large circuits, we output to SBML [29] and imported into COPASI [30].

The merit of seesaw circuits is that it is straightforward to compile them into systems of DNA molecules, with the implicit claim that the experimentally synthesized systems will closely approximate the model ODEs. This claim needs to be tempered by acknowledging a number of additional reactions, not included in our model, that will certainly occur in experimental systems.

First, even without a toehold, a single-stranded DNA domain can displace an identical domain from a double helix, albeit at a rate five or six orders of magnitude slower than *k*_*f*_, the fastest rate for toehold-mediated strand displacement [26–28]. Examples include (i) ‘fuel-gate leak’ where, with reference to figure 1*b*, fuel reacts directly with gate:output, yielding output and gate:fuel even in the absence of input, and (ii) ‘gate–gate leak’ where the single-stranded *S*_3_ domain of gate:output directly interacts with a downstream gate with base strand *T*′*S*_3_*T*′, causing it to release its top strand. So long as leak is not too large, it can be cleaned up with threshold gates.

Second, because we use a universal toehold sequence *T*, *any* signal strand can bind to the toehold of *any* gate complex. However, the uniqueness of the recognition domains *S*_*i*_ ensures that no such binding will lead to branch migration, and the invading signal strand will quickly fall off. While this ‘spurious toehold binding’ will not result in an incorrect strand displacement reaction, it will change the effective reaction rates by temporarily disabling some fraction of gate and threshold complexes and reducing the signal strand concentrations. If all reactions are slowed down to the same degree, then the behaviour changes are inconsequential. Furthermore, the disruption is less at higher temperatures and lower concentrations, and therefore can be avoided at the cost of overall speed.

Third, there is one case where unproductive spurious toehold binding has the potential to cause significantly more disruption. If a signal strand shares only the upstream domain with a threshold complex, it could spuriously bind the entirety of the threshold complex's extended toehold. For example, if the signal strand *S*_1_*TS*_4_ co-existed in a system with the threshold complex of figure 1*c*, it could bind to the 8 nt toehold *s*′_1_*T*′ but could not undergo strand displacement. Such interactions will take significantly longer to resolve, because nucleic acid dissociation rates decrease exponentially with the number (more accurately, free energy) of base pairs that must be broken [31]. As a consequence, these threshold reactions will be slowed down more than gate reactions, decreasing their effectiveness as thresholds. We call this ‘threshold inhibition’. It can be minimized by optimizing toehold lengths, temperature and concentrations.

Fourth, in gates with more than one input, there will be some crosstalk if one or both of the inputs have a threshold. The threshold for the first input can absorb the second input signal strand and visa versa, because both thresholds have the same initial toehold sequence *T*′ even if the extra bases are different. Consequently, after one input exceeds its own threshold, having reacted at a fast rate, it will continue to be absorbed by the other threshold—at roughly the same slower rate with which it reacts with the gate:output complex. We call this ‘threshold crosstalk’. Unlike the previous three problems, threshold crosstalk is intrinsic to the design and thus cannot be reduced by optimizing experimental conditions.

Rather than explicitly add these effects into our model, here we prefer to keep the model as simple as possible, while noting in which circuits we expect that side reactions would have a significant effect. Our main concerns are threshold inhibition and threshold crosstalk. Since threshold inhibition cannot occur in circuits in which every gate immediately upstream of a threshold has exactly one output and no fuel, and threshold crosstalk cannot occur in circuits in which only gates with a single input are allowed to have a threshold, seesaw circuits satisfying both these conditions are called ‘clean’ circuits and we expect them to be well modelled by our simple equations. In principle, any ‘unclean’ circuit may be converted into a ‘clean’ one by inserting a new gate node in the middle of offending wires, moving offending thresholds to the new nodes as necessary. Although the temporal dynamics and equilibrium will not be identical, in many cases the resulting clean circuit will closely approximate the original circuit's behaviour. However, as we will see in the following sections, often the non-idealities of unclean circuits do not significantly disrupt correct behaviour, and conversely it is often possible to find efficient clean circuit implementations directly.

## 4. Feedforward digital circuits

Digital logic has two compelling features for circuit construction: first, it has proved to be very expressive for the synthesis of a wide range of desired behaviours; and second, it is intrinsically robust to a variety of manufacturing and operational defects. The basic principle underlying digital logic is that an intrinsically analogue signal carrier may be considered simply to be either ON or OFF if at each stage of computation, signals are either pushed toward the ideal ON value or pushed toward the ideal OFF value. This is called signal restoration, because if noise or device imperfections slightly corrupt a signal, that deviation from ideal behaviour is cleaned up (perhaps not completely) by subsequent processing without altering the interpretation of the signal as ON or OFF. For example, a digital abstraction might consider signal levels between 0*x* and 0.2*x* as OFF, while signal levels between 0.8*x* and 1*x* are considered ON. Intermediate signal levels—in the transition region—must be transient or else they are considered a fault, because proper behaviour of logic gates is no longer guaranteed for input levels in the intermediate range. Devices that support wider digital abstract ranges are therefore more robust to noise and other imperfections.

In principle, digital logic behaviour can be achieved in analogue mass-action chemistry by exploiting catalysis and non-linearity for signal restoration [32]; catalysis is also essential for ensuring that signal propagation in multi-layer circuits is fast [33]. Seesaw gates easily provide both catalysis and thresholding to implement signal restoration and fast signal propagation. However, once a seesaw gate has been activated and reaches equilibrium, it cannot be re-used, putting a limit on what class of circuits can be implemented.

Feedforward digital circuits are an important class of combinational circuits, i.e. memoryless circuits in which the inputs uniquely determine the outputs without the need to re-use any parts of the circuit. It is well known that any boolean function can be computed (often quite efficiently) by a well-designed feedforward circuit built from AND, OR and NOT gates. Interestingly, with a small cost in circuit size and a minor change in representation, called ‘dual-rail logic’, AND and OR by themselves suffice. We will therefore provide constructions for these basic operations, incorporating signal fan-in, fan-out, routing, and thresholding as may be required to paste the computing gates together into large circuits. We present two schemes for implementing digital logic with seesaw gates. The first scheme, called the ‘1-4 scheme’ because each OR gate requires one seesaw gate and each AND gate requires four seesaw gates, exhibits ideal digital behaviour with large digital abstraction ranges. However, the seesaw circuits are not ‘clean’, and therefore one would expect degraded performance in experimental implementations due to threshold crosstalk and threshold inhibition. The second scheme, called the ‘2-2 scheme’, also exhibits excellent digital behaviour, although with smaller margins. Moreover, the 2-2 scheme circuits are clean, alleviating experimental implementation concerns.

**Figure 3.**
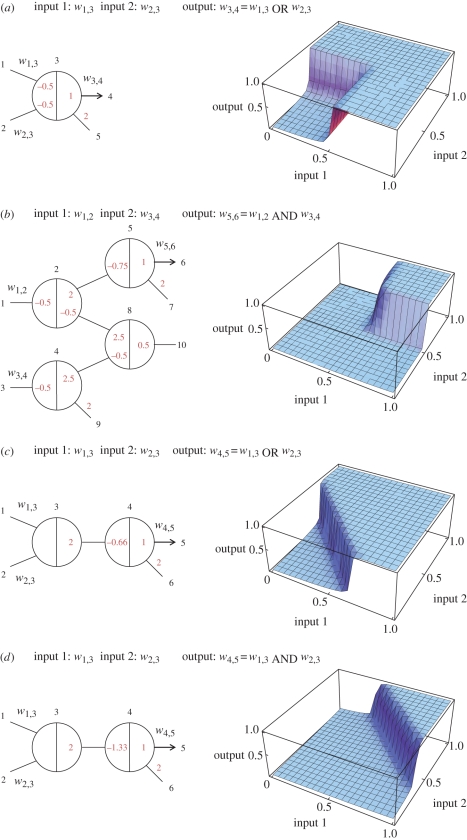
Circuit diagrams and input/output behaviour of boolean logic gates. Output wires with arrowheads indicate that a downstream load is assumed, which consumes signal strands as they are released. (*a*−*b*) A two-input OR gate and a two-input AND gate using, respectively, 1 and 4 seesaw gates, the ‘1-4 scheme’. Circuits constructed using the 1-4 scheme are not clean, and thus would perform worse if threshold crosstalk and threshold inhibition were modelled. (*c*–*d*) A two-input OR gate and a two-input AND gate using two seesaw gates each, the ‘2-2 scheme’. Circuits constructed using the 2-2 scheme are clean. All simulations were performed with the reference concentration 1*x* = 50 nM, and stopped at *t* = 10 h. Here and in all other simulations, *k*_*f*_ = 2 × 10^6^ M^−1^ s^−1^ and *k*_*s*_ = 10^5^ M^−1^ s^−1^.

In the 1-4 scheme, the OR gate (figure 3*a*) is built as a simple extension of the basic catalyst (figure 1*a*) in which there are two input wires, *w*_1,3_ and *w*_2,3_, that each have a threshold. If either exceeds its threshold, it can serve as catalyst for the release onto output *w*_3,4_, driven by exchange with the fuel wire *w*_3,5_. Rather than analyse this subcircuit in isolation, we treat the case where a downstream circuit provides a ‘load’ on the output, in the sense that free output signal strands are irreversibly absorbed, for example by a threshold in the downstream circuit. We plot the total amount of output that is absorbed by the downstream process. With this approach, the behaviour of circuits can be understood without having to calculate exact equilibria: once an input wire exceeds its threshold, catalysis will proceed at some rate until all the output is released. Under the assumption that the threshold reaction rate constant, *k*_*f*_, is 20 times faster than the gate reaction rate constant, *k*_*s*_, the OR gate exhibits ideal digital behaviour in simulations. A digital abstraction with 0–0.4*x* being OFF and 0.6–1*x* being ON is possible. For smaller ratios *k*_*f*_/*k*_*s*_ or shorter times, the sharpness of the threshold would decrease correspondingly.

The AND gate for the 1-4 scheme is a little trickier (figure 3*b*). The idea here is to put two seesaw gates in series (gates 2 and 4), with one gate's fuel being provided by another gate's output—thus gate 2 becomes catalytically active only when both inputs are ON. Gate 8 is a routing device that effectively allows a wire to go from the right side of one gate to the right side of another gate, and gate 5 provides signal restoration to clean up stoichiometric triggering of gate 2 when only its input is ON. Let us verify the four cases for a digital abstraction with 0–0.4*x* being OFF and 0.6–1*x* being ON. First, if *w*_1,2_ and *w*_3,4_ are both OFF, they will be absorbed by their respective thresholds, and nothing more will happen, so the output *w*_5,6_ will remain OFF. Second, if only *w*_3,4_ is ON, then it will drive wire *w*_4,8_ high, which will release the routing catalyst *w*_8,10_ from the routing gate, allowing activity to flow to gate 2's fuel wire, *w*_2,8_. However, with input *w*_1,2_ being OFF, nothing more will happen, and the output will remain OFF. In the third case, both inputs are ON; as before, gate 2's fuel wire is driven high, but now input *w*_1,2_ exceeds its threshold and catalyses activity to flow to wire *w*_2,5_, which in turn exceeds its threshold and catalyses the output to turn ON. In the final case, input *w*_1,2_ is ON but *w*_3,4_ is OFF. In this case, gate 2's fuel *w*_2,8_ remains inactive, but as much as 0.5*x* could be pushed onto *w*_2,5_ from stoichiometric triggering by input *w*_1,2_. However, this does not exceed gate 5's threshold, so the output remains OFF. For this qualitative argument to go through quantitatively, the initial gate and threshold concentrations need to be adjusted carefully; a working choice is shown in figure 3*b*, for which simulations show ideal digital behaviour.

Having established that seesaw circuits can implement digital logic if threshold crosstalk and threshold inhibition are neglected, we set out to find a ‘clean’ implementation where neither issue can arise in an experimental system. This is accomplished by the 2-2 scheme, which can support a digital abstract with 0−0.3*x* being OFF and 0.7−1*x* being ON. In the OR gate (figure 3*c*), the first seesaw gate has two inputs but no threshold and no fuel, thus producing stoichiometric activity on the intermediate wire *w*_3,4_ capable of summing the two inputs. The threshold on the second gate is set at 0.66*x* to be above the maximum sum of two OFF inputs (0.3 + 0.3 = 0.6) and below the minimum sum of an ON input plus an OFF input (0.0 + 0.7 = 0.7). Thus, catalysis can drive the output wire *w*_4,5_ ON only if either or both inputs are ON. Simulations show a sharp threshold along the line [*w*_1,3_]_0_ + [*w*_2,3_]_0_ = 0.66*x*.

The AND gate (figure 3*d*) has the same wiring diagram as the OR gate, but uses a suitably increased threshold. If neither or one input signal is ON, at most 1.3*x* can be pushed onto wire *w*_3,4_, which will be absorbed by the 1.33*x* threshold of gate 4. Only when both input signals are ON, can a signal equal to or greater than 1.4*x* be pushed onto wire *w*_3,4_ and exceed the threshold. Simulations show a sharp threshold along the line [*w*_1,3_]_0_ + [*w*_2,3_]_0_ = 1.33*x*.

Fan-in and fan-out are handled easily in both schemes. More than two inputs (additional fan-in) to an AND/OR gate can be implemented by a binary tree of two-input AND/OR gates. More than one output (fan-out) from an AND/OR gate simply entails connecting more output wires and increasing the concentration of the fuel.

NOT gates appear to be difficult to implement directly. The problem is that a NOT gate must distinguish between a low input signal computed by an upstream gate, in which case it should release its output strand, and an input signal that is low simply because it has not yet been computed, in which case the NOT gate should not do anything yet. If the NOT gate releases its output too soon, and later the input goes high, the damage is done and cannot be undone: downstream gates may have already acted on the NOT gate's output, and those gates cannot turn OFF after they have (prematurely) turned ON.

**Figure 4.**
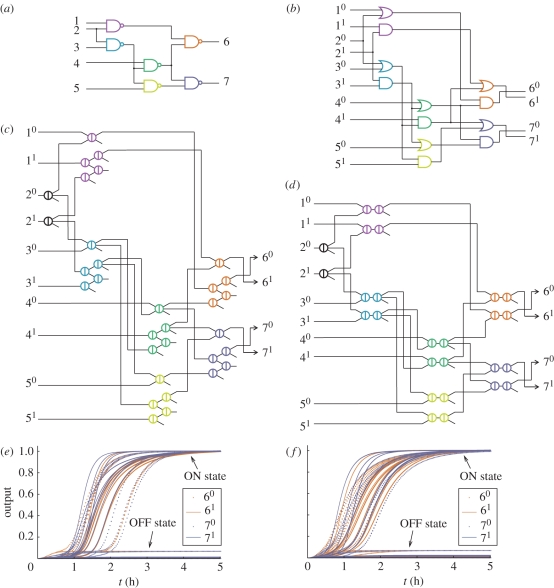
Compiling boolean logic circuits. (*a*) A sample circuit with six gates. (*b*) Translation into an equivalent dual-rail circuit with 12 gates. (*c*,*d*) Translation into an equivalent seesaw gate circuit with 32 gates (1-4 scheme) and 26 gates (2-2 scheme). (*e*,*f*) Simulation results for all 32 possible input vectors in the 1-4 scheme and in the 2-2 scheme. The concentrations of all four dual-rail output species are shown as a function of time. Delays vary with the input, depending the shortest decision path through the network. Simulations were run using the concentration 1*x* = 50 nM, with ON inputs at 0.9*x* and OFF inputs at 0.1*x*. For the 1-4 scheme, the simulated reaction equations were augmented to also model threshold crosstalk, which degrades the performance of OR gates—but the system still works.

To avoid this problem, we use the dual-rail convention [34,35]. We convert a circuit of AND, OR, NOT, NAND, NOR and XOR gates into an equivalent dual-rail circuit that uses only AND and OR, as illustrated in figure 4*a,b* for a circuit of NAND gates. In the new circuit, which will contain roughly twice as many gates, each wire *z* is replaced by two new wires, *z*^0^ and *z*^1^. If neither new wire is ON, this indicates that the logical value of *z* has not been computed yet; if only *z*^0^ is ON, this indicates that the logical value of *z* must be OFF; while if only *z*^1^ is ON, this indicates that the logical value of *z* must be ON. (If both *z*^0^ and *z*^1^ are ON, then the circuit is experiencing a fault.) With this representation, each original AND, OR, NAND, or NOR gate can be implemented using one AND gate and one OR gate; XOR requires four OR gates and two AND gates; and a NOT gate simply requires rerouting wires and swapping their labels. For example, the original gate *z* = *x* NAND *y* becomes the two gates, *z*^1^ = *x*^0^ OR *y*^0^ and *z*^0^ = *x*^1^ AND *y*^1^, which can be verified by inspection. Furthermore, dual-rail logic effectively solves the problem of NOT gates because no computation will take place before the input signals arrive.

To demonstrate the process of making feedforward digital logic circuits out of seesaw gates, we translated a six NAND gate circuit (figure 4*a*) to its equivalent dual-rail circuit (figure 4*b*), and then to its equivalent seesaw circuits using both the 1-4 scheme (figure 4*c*) and the 2-2 scheme (figure 4*d*). Extra fan-out gates were introduced for inputs that were used more than once, and a downstream load was included to pull the output high. The corresponding system of ODEs describing the network's mass action kinetics was then simulated for all 32 possible input combinations. As shown in figure 4*e,f*, in every case both outputs reached either a clear OFF or ON concentration level, which was verified to be correct even with imperfect input concentrations. Even for the unclean circuit (figure 4*e*) simulated with threshold crosstalk equations included, the imperfect behaviour of each logic operation was greatly improved by the signal restoration built into the downstream operations, and correct digital function was observed. This provides concrete evidence that the digital logic circuits compose well.

**Figure 5.**
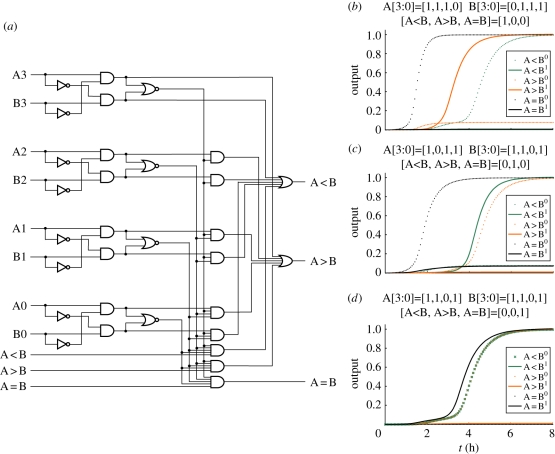
A 74L85 standard 4-bit magnitude comparator (four layers deep) and its seesaw circuit simulation, with 1*x* = 50 nM. (*a*) The digital logic circuit diagram. The corresponding seesaw circuit has roughly 100 seesaw gates. (*b*) Seesaw circuit simulation with selected input vector of A greater than B. (*c*) Seesaw circuit simulation with selected input vector of *A* smaller than *B*. (*d*) Seesaw circuit simulation with selected input vector of A equal to B.

With realistic rate constants, our simulations suggest that the timescales for seesaw circuits are the order of an hour per layer of digital logic. This being a bit slow, it is worth noting that there are interesting computations that require not too many layers. For example, a standard 74L85 4-bit magnitude comparator, with roughly 30 logic gates, requires only four layers. We compiled this circuit to seesaw gates using the 2-2 scheme, and ODE simulations demonstrated correct behaviour (figure 5).

## 5. Relay contact circuits

In his seminal Master's thesis [36], Claude Shannon established a systematic symbolic approach to the analysis and design of digital circuits. A prevalent technology at the time was relay contact circuits, in which input switching signals (A, B, C, etc.) opened or closed electrical contacts in a network, either allowing current to flow through the network, or not. Like circuits made of AND, OR, and NOT gates, relay contact circuits can concisely implement arbitrary boolean functions. To illustrate the flexibility of the seesaw gate motif, we provide a general method to compile relay contact circuits down to equivalent seesaw gate circuits. (Here, we consider only circuits where relays are directly controlled by external input signals. Using the output current signal of a relay circuit as the input switching signal to another relay circuit is left as an exercise to the reader.)

**Figure 6.**
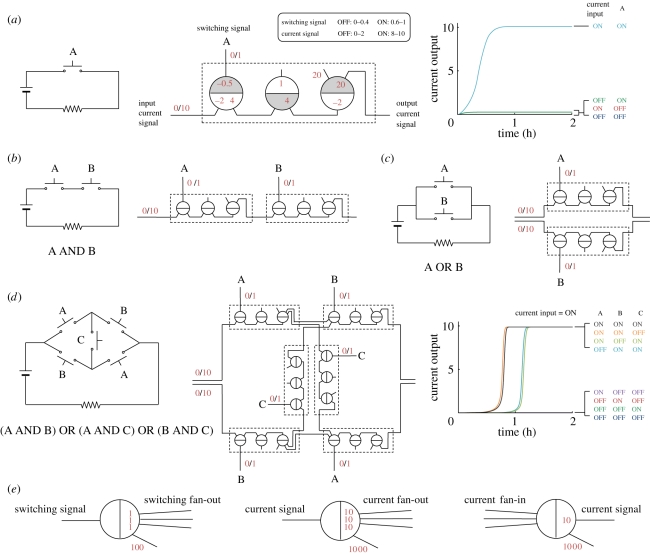
Implementation of relay circuits. (*a*) A simple circuit with current source (battery) and controlled device (denoted by a resistor), the corresponding seesaw gate circuit, and its simulation using 1*x* = 50 nM. Shaded and unshaded sides of seesaw gates assist checking that a wire always connects different sides of two seesaw gates as required by node polarity, i.e. each wire connects the shaded side of one seesaw gate to the unshaded side of another. Switching signal *A* is provided at 1*x* if ON, or else 0.1*x* if OFF. Input current signal was provided at 10*x*; to verify that no output signal is produced when the current input is OFF, a 1*x* signal was provided. (*b*) AND logic. (*c*) OR logic. (*d*) A more complex circuit. Overlapping trajectories (orange and light blue) were shifted to the left by 100 s to make them visible. (*e*) Switching signal fan-out, current signal fan-out and current signal fan-in.

The basic primitives for constructing relay contact circuits are simple. The function of a relay contact switch (figure 6*a*, left) is similar to that of a single seesaw gate configured as a catalyst: current signal flows only if the switching signal is ON. However, for a modular implementation that maintains the orientation and intensity of the current flow, we use three seesaw gates to implement each relay contact switch (figure 6*a*, middle). The input current signal arrives on the wire at the left, the switching signal provides catalytic input to the leftmost seesaw gate, and after a chain of events, the output current signal goes high if and only if the switching signal A is ON. The thresholds help to clean up any leak in the current signal or switching signal. (For example, when the switching signal is ON but the current input is OFF, a small amount of signal will still be produced on the wire that connects the leftmost and the middle seesaw gates, due to stoichiometric triggering.) The middle of the three seesaw gates is a routing gate added to accommodate the intrinsic polarity of seesaw gates, so that the output current signal of one relay can be directly used as the input current signal to another (as can be easily verified using the shading of gate nodes in figure 6*a*). The rightmost seesaw gate acts as a signal restorer that pushes the current signal to the standard ON/OFF levels, compensating for input current signal decay that is a consequence of its role as a fuel. (Consider a catalytic gate without downstream load, as in figure 1*a*: at equilibrium, the output signal level is strictly less than the initial fuel level.) The simulation of this circuit shows that only when the current signal is ON and the switching signal A is ON, can the output signal reach an ON state (figure 6*a*, right).

More complex regulatory logic can be implemented by composing relay contact subcircuits. Two switches (or subcircuits) in series perform an AND operation (figure 6*b*). Two switches (or subcircuits) in parallel perform an OR operation (figure 6*c*). The only innovation here is that two distinct current input and output wires are used. Fan-out of the current input (figure 6*e*, middle) can provide such a signal, and fan-in of the current output (figure 6*e*, right) can consolidate the signal into a single wire, if desired.

In general, any relay contact circuit can be compiled down to seesaw gates, with each relay contact implemented using the three gate scheme. Since the current flow direction on a wire in the relay contact circuit may be unknown (e.g. the wire connected by switch C in figure 6*d*, left), but the seesaw implementation is directional, in such cases two triples of seesaw gates are needed for each relay contact. However, after the power supply is added, only some of the directions will be active. A more complex circuit (figure 6*d*) was automatically compiled to seesaw gates in this manner. Besides current signal fan-out, current signal fan-in, we also need switching signal fan-out (figure 6*e*, left) to produce different switching signal strands for different seesaw gates representing the same logical input (such as the multiple instances of A and B in figure 6*d*).

## 6. Analogue time-domain circuits

The behaviour of seesaw gate circuits is intrinsically analogue. Following the approach of Zhang *et al.* [3], we construct amplifier cascades with initial quadratic growth and with initial exponential growth. More complex temporal dynamics can also be synthesized, such as a pulse generator.

**Figure 7.**
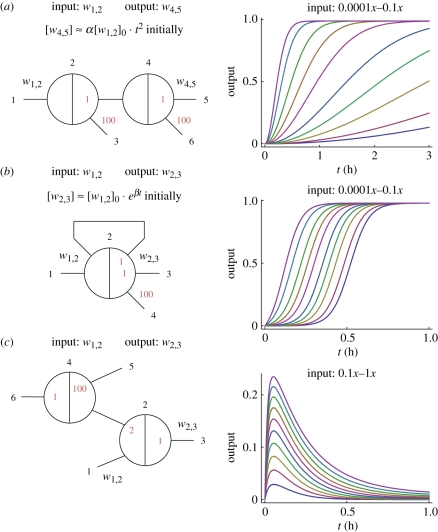
Analogue time-domain circuits. (*a*) A catalytic cascade that exhibits initially quadratic growth, with *α* ≈ 125 h^−2^. Temporal trajectories are shown for a series of exponentially decreasing initial input concentrations. (*b*) A positive feedback circuit that exhibits initially exponential growth, with *β* ≈ 17 h^−1^. The same series of exponentially decreasing input concentrations now yields a series of trajectories with linearly increasing half-completion times. (*c*) A pulse-generating circuit. Pulse amplitude depends on the input concentration. Here, we use a linear series of input concentrations between 0*x* and 1*x*. All simulations use 1*x* = 50 nM.

The amplifier shown in figure 7*a* is a two-stage feedforward cascade. Input signal *w*_1,2_ catalytically pushes strands onto wire *w*_2,4_, which exhibits initially linear growth with time. Signal *w*_2,4_ also serves as a catalyst for the release of output strand *w*_4,5_, which therefore initially grows quadratically with time. While the output remains below 0.5*x*, a good approximation is [*w*_4,5_] ≈ *α* [*w*_1,2_]_0_.*t*^2^ with *α* ≈125 h^−2^.

The amplifier shown in figure 7*b* is a one-stage feedback cascade. Initially, input signal *w*_1,2_ catalytically releases strands onto both the feedback wire *w*_2,2_ and the output wire *w*_2,3_. However, signal strand *w*_2,2_ contains the recognition domain of gate 2 on both its left side and its right side, so therefore once it has been released, it can play the role of the input signal in catalysing output of more *w*_2,2_ and *w*_2,3_ from the gate. Because strand *w*_2,2_ is catalytically active in releasing additional copies of itself, its concentration increases exponentially (until gate concentrations drop to low levels). The output strand *w*_2,3_, being released at the same rate as the feedback strand, also grows roughly exponentially. We found that for small inputs, [*w*_2,3_] ≈ [*w*_1,2_]_0_.*e*^*β*t^ with *β* ≈ 17 h^−1^.

The pulse generator shown in figure 7*c* illustrates a non-amplifying temporal dynamic. The basic idea is that the input strand *w*_1,2_ initially releases a significant amount of output *w*_2,3_, but later this strand is pulled back into gate 2 as gate 4 becomes active. Why does this happen? Free *w*_2,3_ can stoichiometrically trigger release of *w*_4,2_, which is now entropically sucked into gate 4 as the catalyst *w*_6,4_ is activated and equilibrium is established. The wire/gate ratio *r*_4_ cannot exceed 1/99 for wire *w*_4,5_; this same ratio must apply to wire *w*_4,2_, with the result that almost all the free signal strand will be absorbed by gate 4. Thus, at equilibrium [*g*_4:4,2_] ≈ 0.99*x* ≈ [*g*_4,2:2_] and [*w*_4,2_] ≈ 0.01*x*. So the wire/gate ratio *r*_2_ must also be low, which in turn pulls *w*_2,3_ back into gate 2, similarly pulling the output low. (Effectively, gate 4 behaves like a threshold complex, and could be replaced by one if desired.)

These three simple examples make it clear that seesaw circuits can support interesting dynamical behaviours, even without using explicit threshold complexes. Further examples could be given, for example, adding thresholds to the circuit in figure 7*a* and extending it in series would yield a delay line, from which fan-out wires could provide signals that turn ON at specified times after the input arrives. Characterizing the full range of analogue behaviours that can be achieved, with and without threshold complexes, is an important open question.

## 7. Discussion

This project was inspired by the remarkable success of scaffolded DNA origami [20,37,38] for programming the self-assembly of hundreds of DNA strands into a single target structure. The self-assembly of DNA origami is extraordinarily reliable despite that DNA sequences cannot be optimized to avoid undesired binding interactions and that unpurified DNA strands are used, implying that the system is surprisingly robust to spurious binding, to imprecisely known strand concentrations, and to subpopulations of incomplete or damaged molecules. We were therefore looking for an analogous design for DNA strand displacement-based circuits—one that would require minimal sequence design effort and work well even with unpurified strands and unreliable concentrations. Does our proposed seesaw gate motif live up to our hopes and expectations as a DNA circuit component suitable for scaling up to large and complex circuits? We see some encouraging features, some concerns and some clear challenges.

First, design of large feedforward digital circuits looks promising. At the highest level, abstract specifications for circuit function can be expressed concisely using existing hardware description languages such as Verilog [39,40] and VHDL [41], then compiled down to a gate level netlist specifying elementary gates (AND, OR, NOT, NOR, NAND, XOR) and their connectivity. Thus, the sheer complexity of large-scale circuit design can be managed by off-the-shelf tools. The next step is compiling the digital logic netlist down to the seesaw gate circuit abstraction, using the constructions described above for dual-rail logic. This is straightforward if no circuit size optimizations are attempted. To achieve the final step of designing molecules, we must assign sequences to each gate base strand. For this purpose, a single large set of sufficiently distinct domain sequences would enable construction of any circuit containing up to the given number of seesaw gates.

Seesaw circuits make use of two kinds of sequence domains: long recognition domains and short toeholds. In principle, multiple distinct toehold sequences could be used, which would reduce the spurious toehold binding problem. However, the toehold length is severely constrained by physical factors, and for a fixed length there are a limited number of sufficiently distinct sequences. With the goal of a scalable architecture in mind, we thought it was simplest to confront this limitation early on, and we adopted the universal toehold scheme, understanding that to mitigate the effects of unproductive spurious binding, the 1*x* standard concentration may have to be lowered as circuits get larger. Thus, the burden of making reactions highly specific falls to the recognition domains.

Can we design sufficiently many recognition domain sequences to scale up to circuits with many gates? We consider two design criteria: (i) signal strands should not have strong secondary structure and should not interact with each other, and (ii) strand displacement should be unable to proceed if the invading sequence is not the desired signal strand. The first criterion can be satisfied by standard DNA sequence design methods [42,43]; here we take the easy approach by using a three letter code (A, C and T) for the signal strands, thus ensuring that problematic secondary structures and interactions are unlikely [44–46]. The gate base strand will therefore consist of (A, G, and T). Because of the complete independence of domains within the seesaw gate motif, no system-level conflicts arise when strand sequences are generated by concatenation. The second criterion may be formalized as combinatorial sequence constraints. For example, we could require that at least 30 per cent of bases are different for any two distinct recognition domains; as each mismatch impedes branch migration speed by a factor of roughly 10 [47,48], even five mismatches will dramatically reduce crosstalk. Additionally, we require that mismatches are spread out, so that when the wrong signal strand interacts with a gate, it will quickly encounter difficulties and dissociate; specifically, the longest run of matches must be less than 35 per cent of the domain length. Finally, to reduce synthesis errors and ensure comparable melting temperatures, we require that there are no more than four A's or T's in a row, no more than three C's or G's in a row, and that sequences have between 30 and 70 per cent GC-content (cf. constraints 1,7,8 of [49]). Using a ‘sphere-packing’ technique [50], we have found sets of sizes 67, 399, 3097 and 8343 for recognition domains of lengths 10, 15, 20 and 25, respectively, confirming the theoretically expected exponential growth in available sequence space (empirically, *N* ≈ 1.5 × 2^*L*/2^). This is enough to construct some interesting circuits. The caveat is that while reasonable, the criteria used here are ad hoc; we are not certain that molecules designed using these criteria will work consistently in the laboratory, and additional or alternative design criteria may need to be articulated.

**Figure 8.**
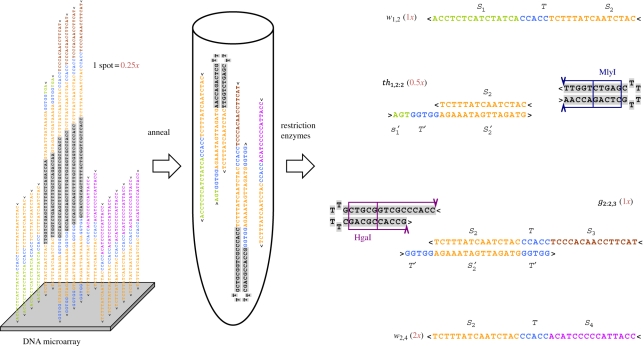
Parallel preparation of seesaw gates as hairpin precursors. Using DNA microarray synthesis technologies, each gate, threshold and fuel is made as a single strand. After cleavage from the surface, the mixture is annealed to form hairpins. Restriction enzymes then cleave the hairpins to form gate and threshold complexes. Relative concentrations (red numbers) are set based on the number of DNA chip spots dedicated to a given strand; in this example, one spot produces 0.25*x*.

Can so many distinct sequences be synthesized and handled in the laboratory? A substantial difficulty with our previous work [6,3,18] was that each gate molecule was a complex of multiple strands that had to be separately annealed together, and each complex had to be purified to remove excess single-stranded species and malformed gate substrates. In the case of seesaw gates, an additional difficulty is presented by self-loop gates such as *g*_2,2:2_ of figure 7*b*, for which straightforward annealing would yield an equal amount of the undesirable *g*_2:2,2_; instead, a multi-step purification procedure would be necessary. Thankfully, the simplicity of the seesaw gate motif makes laboratory procedures for synthesizing gates and circuits plausible to carry out on a large scale in a manner that avoids this problem. Here, we aim to simultaneously prepare all gate complexes together in a single test tube; to do so, we must ensure that different gate species do not interact, and that the strands needed to form a given gate complex find each other efficiently. For our solution, we draw inspiration from the observation [51,52] that mixtures of hairpin molecules, when annealed, are likely to form non-interacting intramolecular hairpins even if at room temperature there exist lower free energy states involving intermolecular complexes. This occurs because the intramolecular hairpins are typically stable at some moderately high temperature, above the melting temperature of the intermolecular complexes—thus, during annealing, the hairpins form first and become kinetically trapped. The implication for gates is that if each gate species can be synthesized initially as a hairpin precursor, annealing all such gate precursors in a single reaction will result in a high yield of properly formed non-interacting molecules. Figure 8 shows our realization of this scheme. After annealing, incubation with appropriate restriction enzymes removes the now-undesired linker subsequence, resulting in a well-formed complex of two strands. The entire solution could be purified by gel, since all gates are the same size; all threshold gates could be purified similarly. One way or another, making circuit function robust to sloppy parallel gate preparation methods will be crucial to scaling up existing DNA circuits to hundreds or thousands of gates.

The hairpin gate precursor architecture also facilitates the synthesis of the DNA strands themselves. We envision all hairpin strands being synthesized in parallel on a DNA microarray, such as those offered by Agilent and NimbleGen that are currently capable of synthesizing approximately 10^6^ distinct sequences with lengths approximately 50 nt in quantities of approximately 10^6^ molecules per spot [53]. With 5 nt toeholds and 15 nt recognition domains, our hairpins would be up to 91 nt, while with 25 nt recognition domains our hairpins would be up to 121 nt, which appears within reach using new micro array technologies [54]. To estimate the circuit complexity that could be synthesized, consider the 2-2 scheme for digital logic circuits. If each spot provides a 0.33*x* concentration in our chosen reaction volume (this is the minimal increment we use in the 2-2 scheme), then synthesizing the material for an AND or OR gate (which on average require 6*x*) would require the use of 18 spots. This corresponds to approximately 50 000 gates or 5000 gates if we want 10 times more molecules of each species. After synthesis, linkers attaching strands to the slide can be cleaved, and a mixture of all strands can be collected in a single tube, annealed to form hairpins, digested with restriction enzymes to produce gates, and then gel-purified to eliminate non-functional molecules (cf. [55]). Thus, all molecules for the entire circuit are synthesized and processed in parallel in a single tube.

Once designed and synthesized, will the DNA circuits work? The first question is speed. If the maximum total concentration for reliable DNA gate operation is 100 µM (perhaps optimistic), then 1*x* would be 3 nM for a 5000 logic gate circuit, and the slowest reaction half-times (the effective gate delay) would be roughly one day. If this is considered too slow, either one must resign oneself to smaller circuits, for which the concentrations can be higher, or one must find a way to speed up hybridization reactions in dilute complex mixtures. For example, the phenol emulsion reassociation technique (PERT) has been reported to speed-up hybridization dynamics by four orders of magnitude [56,57]. If the exponential dependence on toehold lengths is preserved under these conditions, this would reduce the gate delay to roughly 10 seconds.

The second question is whether the computation will be correct. For feedforward digital circuits, thresholding and signal restoration (the digital abstraction) is expected to provide some robustness to variations in concentrations, to leak, and to minor crosstalk. However, experimental exploration of seesaw gate circuits will be needed to evaluate the potential for producing reliable function in practice. We anticipate that there will be many unforeseen difficulties. In summary, we have proposed a new catalytic DNA gate that appears to be suitable for scaling up to analogue and digital circuits incorporating thousands of gates with a reasonable expectation that adequate speed and reliability could be achieved.

However, a limitation of this work is that the circuit constructions we described all function by completely depleting key gate and fuel species, hence each circuit preparation can be used only once. This prevents us from implementing sequential circuits containing buffers, flip-flops, resets and clocks that orchestrate the re-use of circuit elements and can process time-varying input signals. We do not know at this point whether this limitation is essential to the seesaw gate motif. Because threshold complexes function by being completely consumed, it appears that this question is related to characterizing the behaviours possible in seesaw circuits without thresholds. Especially interesting is how the equilibrium concentrations within such circuits respond to changes in total signal placed on input/output wires. Similarly, we do not yet have a characterization of the class of analogue dynamics that can be achieved in the use-once setting, although it appears to be a rich space of behaviours. In considering what can be achieved with the seesaw motif, one might be tempted to add additional molecular mechanisms in order to obtain new behaviours; however, one must keep in mind that fully general digital, analogue, and algorithmic behaviours have already been shown to be possible using more complex multi-stranded gate complexes [18,58]. Unfortunately, such gates are more difficult to prepare experimentally; the value of the seesaw motif lies in its simplicity and experimental tractability.

It is not yet clear what applications seesaw circuits will find in practice. However, other DNA and RNA reaction cascades based on toehold-mediated strand displacement have already been demonstrated successfully as programmable cancer drugs that work *in vivo* [59], and as programmable amplification mechanisms for improving *in situ* fluorescence imaging of mRNA [60]. While the cellular environment may interfere with proper seesaw circuit function, it is not implausible that seesaw circuits could be adapted or modified to work in that context. Already, the entropy-driven toehold-exchange catalytic mechanism used in seesaw circuits has been investigated as an amplifier for *in vitro* biomedical diagnostics [61]. Additional input and output interfaces between nucleic acid circuits and other chemical species would allow nucleic acid circuits to serve as embedded controllers for a wide range of molecular events. Finally, the simplicity and power of the basic seesaw architecture makes one wonder whether circuits based on RNA toehold exchange processes could exist in biological systems. While it may be a superficial similarity between seesaw gates and miRNAs and siRNAs [62,63], all of which are short duplex nucleic acids with single-stranded overhangs that arise by cleavage of hairpin precursors, it is harder to imagine biological systems avoiding than exploiting seesaw-like mechanisms.
